# LSTM-based ensemble models for keystroke dynamics authentication: integrating explainable AI for transparency

**DOI:** 10.3389/frai.2026.1860248

**Published:** 2026-07-16

**Authors:** K. Sasikumar, Sivakumar Nagarajan

**Affiliations:** School of Computer Science and Engineering, Vellore Institute of Technology, Vellore, Tamil Nadu, India

**Keywords:** behavioral biometrics, ensemble learning, explainable AI (XAI), keystroke dynamics, LSTM, user authentication

## Abstract

The increasing insecurity of traditional methods such as passwords and PINs has raised significant interest in behavioral biometrics. Keystroke Dynamics (KSD), which relies on the unique manner in which an individual types, is a promising candidate for continuous and unobtrusive authentication. This study presents a hybrid model for KSD that combines a Long Short-Term Memory (LSTM) network with an ensemble of Random Forest, XGBoost, and Multilayer Perceptron classifiers using a soft-voting strategy. The model incorporates advanced feature engineering techniques to capture meaningful typing patterns and user-specific behavioral characteristics. The temporal features extracted by the LSTM are effectively classified by the ensemble model, resulting in strong authentication performance on the KDA Keystroke Dynamics dataset. The proposed approach achieved an accuracy of 94.75%, with a recall of 94.89%, precision of 94.75%, F1-score of 94.76%, and MCC of 94.65%. Furthermore, the model demonstrated efficient real-time performance with a throughput of 16,092.93 samples per second and a detection time of only 0.0621 ms per sample. In addition, SHAP-based explainability analysis enhanced model interpretability by identifying the most influential keystroke features contributing to authentication decisions. Overall, the proposed framework provides a secure, scalable, interpretable, and computationally efficient solution for real-time keystroke dynamics-based user authentication.

## 1 Introduction

In the modern interconnected digital world, cyberattacks are more frequent and sophisticated than ever before. The frequency and severity of cyberattacks have increased significantly in recent years (Admass et al., 2023). The healthcare sector continues to experience the highest average cost of data breaches, exceeding $9 million, while financial, industrial, and technology sectors also face substantial economic losses due to cyber incidents (IBM, 2024). The global cost of cybercrime is projected to reach $10.5 trillion by 2025, with more than 1,100 breaches affecting approximately 320 million records by 2023. Phishing attacks increased by 61%, ransomware incidents increased by 82.7%, and brute-force RDP attacks increased by 400%, emphasizing the need for stronger cybersecurity measures. Credentials and PINs remain the most commonly used authentication methods; however, their effectiveness has declined due to weak or reused passwords and unsafe user practices, creating a need for more secure and reliable authentication mechanisms (Bhana and Flowerday, 2020).

Behavioral biometrics, particularly keystroke dynamics, have emerged as feasible alternatives to modern cybersecurity. Keystroke dynamics looks at typing patterns, such as keystroke duration, delay, and flight time, to determine user identification in a non-intrusive and continuous capacity. These methods are different from traditional biometrics like fingerprints or facial recognition, as they run in the background and do not require extra hardware. Besides, typing patterns are characteristic of individuals, thus they are very hard to duplicate and forge. This leads to continuous authentication meaning that the identity is still verified even after the authentication process is complete and also the initial log-in. In this way, the sponsorship of another behavioral layer to the authentication system not only secures the system but also minimizes the risk of stolen credentials (Tsimperidis et al., 2021a; Quimatio et al., 2022). Keystroke dynamics takes odd typing patterns and uses them as a definitive key for unlocking the computer, thus, this technology is both inexpensive and very secure comparable to the usage of standard keyboards. Keystroke dynamics can be relied upon as a viable option for traditional authentication methods because they reduce the risk of continuous authentication, such session hijacking and system access. However, the success of the keystroke dynamics is based on the accuracy of the classification methods used. The issue of typing variability is heightened the when classification methods require high levels of accuracy (Crawford, 2010).

In this study, complex feature engineering techniques were applied to generate and refine features from keystroke dynamic data, there by optimizing the value of the information input to the AI models. Next, the features that resulted from the process were forwarded to an LSTM Network which was supposed to capture the high-level temporal representation of the data. The output of the LSTM Network was passed into an ensemble classifier for the final classification, which ensured a higher accuracy and robustness of the decision. Explainable Artificial Intelligence (XAI) has been deployed at the same time to enhance the system's transparency, explainability, and trust in the higher reliability of the keystroke dynamics authentication systems while providing richer insights into the model's decision making and reducing bias. The key contributions of this research work can be summarized as follows:

- Create an LSTM based ensemble model combined with advanced feature engineering to effectively quantify the rich typing dynamics.
- Incorporate Random Forest, XGBoost, and MLP classifiers with soft voting to attain robust and accurate classification.
- Include Explainable AI (XAI) to provide transparency and understanding to the decision-making process of the model.
- It achieves high computational efficiency and real-time applicability for creating safe and scalable behavioral biometric authentication.

### 1.1 Structure of the paper

The remainder of this paper is organized as follows. Section 2 reviews the existing literature on keystroke dynamics, behavioral biometrics, and authentication techniques. Section 3 presents the proposed authentication framework, including the keystroke dynamics dataset, data preprocessing, feature engineering, and model development. Section 4 describes the experimental setup, evaluation metrics, and performance analysis. Section 5 discusses model interpretability using Explainable Artificial Intelligence (XAI) techniques. Finally, Section 6 summarizes the main findings, contributions, limitations, and future research directions.

## 2 Related work

Keystroke behavior is an authentication technique that authenticates a person based on their typing pattern. This is different from traditional password mechanisms as it offers a different type of protection as the system verifies how a user types rather than what a user types (Porwik et al., 2020). There are two basic types of keystroke dynamics systems: fixed-text authentication (Altwaijry, 2020; Mhenni et al., 2019; Al-Obaidi and Al-Jarrah, 2016), in which a user provides a set text, such as a password or a PIN, and free-text authentication in which the typing behavior is assessed based on unlimited text. Fixed text authentication is generally used for a first-time login, while free-text authentication (Kim and Kang, 2020; Ayotte et al., 2020; Tsai and Shih, 2019) provides verification of a user while continuously using the system as it provides greater security while the user is using the system. The following sections explore the primary research studies on keystroke dynamics, focusing on the objectives, methods, datasets, and results, to present an in-depth overview of the progress and challenges in this field.

Tsimperidis et al. (2018) examined the use of keystroke dynamics to detect gender and achieved an accuracy of 95.6%, by applying feature selection techniques and using an SVM and random forest on a new dataset. However, it has many limitations, including a small dataset, focus on gender classification, and heavy use of feature selection, which can limit the utility of the results. In addition, issues concerning scalability and real-time applications connected to privacy, indicate that the future of keystroke dynamics will be discovered only if larger datasets and more efficient methods are employed. Muliono et al. (2018) applied machine learning and deep learning to investigate the keystroke dynamics in user authentication. They checked the SVM against deep learning models using a dataset of 20,400 samples from 51 participants, and it was shown that deep learning with the Nadam optimizer achieved 92.60% accuracy. However, the analysis is limited by the use of only three features and a single dataset, which limits its generalizability and scalability.

Jawed et al. (2018) utilized keystroke and tap dynamics to detect anomalies using CyberSleep, achieving 99% accuracy on Android and 90% accuracy on Windows using an OCSVM and multiclass classifiers. It highlights the potential, but has poor robustness owing to its feature concentration. Piugie et al. (2022) used deep learning methodologies to investigate the application of keystroke dynamics to identify users by converting time-series data into 3D images that can be classified using CNNs. Deep learning methodologies were used to investigate the application of keystroke dynamics to identify users by converting time-series data into 3D images that can be classified with CNNs utilizing the GREYC-NISLAB dataset to test six pre-trained models, including ResNet-101 and GoogleNet, and found that GoogleNet achieved the best EER of 4.89%. However, this study's lack of real-time applicability and scalability, along with its exclusive focus on predefined passphrases, limit its potential for widespread adoption.

Salem et al. (2019) presented a keystroke dynamics (KSD) authentication approach for mobile security that utilizes a software keyboard to capture timing and non-timing characteristics, such as keystroke press duration and touch pressure. Machine learning models, including Random Forest, obtained an EER of 0.45% and thus became the top models. Although it is the best model in terms of accuracy, it has a major drawback, its applicability to dynamic typing scenarios due to its dependence on static text. User recognition through keystrokes using Differential Evolution was the focus of research in Wu et al. (2019). The results showed that holding and UD time features together gave the best results, with a 2.62% increase in accuracy when using Random Forest on the RHU dataset. Limitations such as very small datasets, the significance of the mentioned temporary features, and scalability were pointed out in this study.

Kim et al. (2020) introduced a feature selection technique for keystroke dynamics authentication that made use of statistical measures, such as trimmed mean and coefficients of variation, leading to a remarkable reduction in error rates by 21.8%. This technique was able to provide moderate accuracy in the case of a smartphone dataset but only a limited number of subjects were included in the study, and the focus was restricted only to PIN-based user activity, and not to testing the proposed method in real-time applications. Darabseh and Pal (2020) explored keystroke dynamics using a dataset of 28 users and evaluate the performance of 18 classifiers. The Random Forest model out of the three highest has an accuracy of 91%, where as the HBOS model is superior to the other one-class models with an accuracy of 81%. However, the results can be considered promising for sectors in which no impostor data are available. The study concentrated on temporal features only and did not evaluate the real-time performance. Sahu et al. (2020) proposed a method based on distance for keystroke classification of multiple users that integrates the scaled Manhattan distance, PCA/Kernel-PCA, and Ordinal UNLOC. By applying the CMU keystroke dataset with a maximum of 200 samples, the technique achieved a peak accuracy of 87.5% with Kernel-PCA and Ordinal UNLOC, thereby surpassing the traditional PCA and nearest neighbor methods. Despite these remarkable results, the research was constrained by the use of predefined passwords, problems with scalability, and no real-time assessment.

Chang et al. (2022) applied a CNN-GRU deep learning model for user identification using free-text keystroke dynamics. The model trained on the Buffalo dataset containing 148 participants achieved an accuracy of 94.6% and an EER of 0.0386, which is superior to traditional techniques. The model, which is powerful and scalable, relies substantially on the Buffalo dataset and particular timing characteristics, making it less versatile for other datasets and typing conditions. Tsimperidis et al. (2021b) used a set of 387 typing samples to categorize keyboard dynamics into gender and age groups, which consisted of variables, such as keystroke lengths and down-down (DD) diagram latencies. It employs five classifiers, including SVM and Radial Basis Function Networks (RBFN), with the RBFN yielding a commendable accuracy of 78.3%. The dataset was small and unbalanced, which may have affected its representativeness. The authors of this study (Choi et al., 2021) proposed a system for authentication through keystroke dynamics relying on the use of unique keypads programmed using the Mersenne Twister algorithm. The performance of the Manhattan distance-based classifier was tested on data collected from 13 individuals over four keypads and with 152 features, achieving the highest EER of 9.64%. The reliance on fixed six-digit PINs hinders their use in dynamic typing.

Quimatio et al. (2022) developed a keystroke dynamics authentication method using bagging ensembles with SVM, KNN, and decision tree classifiers. They tested the approach using a CMU dataset, that included 51 users, and reported an accuracy of 95.65%. The feature set consisted of Hold Time and DD Time, and the process of hyperparameter tuning was employed to boost the performance. Nevertheless, this study had two main limitations: it was based on fixed passwords and had a small dataset. Wang et al. (2022) proposed a deep learning-based system that used keystroke dynamics authentication with LSTM and CNN to capture timing parameters such as Hold Time and Latency. The model performed better than the conventional methods on the two datasets, reaching accuracies of 98.2% and 96.5%, respectively; however, the combination of LSTM and CNN made it very complicated.

Altwaijry (2023) used data from 85 users, who were either English or Arabic speakers, to analyze how the language being typed influenced keystroke dynamics. The outcome of the research was that the language used determined the typing patterns more than the keyboard layout, and the same-language testing accuracy was 99% but it dropped to 50%–54% in cross-language testing. Grunova and Tsimperidis (2023) used keyboard dynamics to determine the age and educational level of Bulgarian speakers. By executing a classification based on age, this study achieved an accuracy of 93.48% with Random Forest, where that based on education reached an accuracy of 86.96% with random forest and multilayer perceptron. Although based on a small dataset, the current study demonstrates the potential of keystroke dynamics in demographic analyses.

Yang et al. (2024) proposed a method for cross-device keystroke authentication based on federated learning, and achieved an accuracy of 85%–90% through the extraction of common features such as timing and activity data. The model is able to perform consistently well over different devices while also ensuring privacy, but at the same time, it needs to be further developed in order to adjust to the variations in user behavior. The research conducted by Tsimperidis et al. (2024) led to the creation of the IKDD dataset, which consisted of 1.85 million keystrokes from 164 people in total. In addition, the dataset contains demographic data that are connected to the participants, such as gender, age, handedness, mother tongue, and education. In terms of user classification, the accuracy values were 81.2% for gender (using RBFN) and 70.5% for age (using SVM). The dataset can record real typing behaviors while still providing adequate privacy protection, thus increasing its applicability in both biometrics and user profiling research. Shadman et al. (2025) presented a comprehensive survey of keystroke dynamics, covering datasets, authentication algorithms, feature engineering methods, mobile/touchscreen authentication, and real-world applications. The study also discussed important challenges such as data privacy, device variation, user behavior changes, and synthetic forgery attacks. However, as a survey paper, it did not propose or experimentally validate a new authentication model. An overview of previous studies on user authentication and classification based on keystroke dynamics is presented in Table 1.

**Table 1:** Overview of related work on keystroke dynamics-based authentication.

Year & objective	Algorithms	Dataset	Technical contributions and limitations
2023 (Altwaijry, 2023) – Impact of typing language on keystroke dynamics	AdaBoost, DT, RF, SVM	Bilingual keystroke dynamics dataset	Showed that typing behavior changes across languages; separate language profiles may be needed for better authentication.
2023 (Grunova and Tsimperidis, 2023) – Age and education-level classification	Naïve Bayes, SVM, MLP, RF	Bulgarian free-text keystroke dataset	Random Forest gave good classification results; however, the study used a relatively small dataset.
2024 (Yang et al., 2024) – Cross-device free-text authentication	Federated learning, CNN	Dhakal dataset	Supported authentication across different devices while preserving privacy; performance varied across device types.
2024 (Tsimperidis et al., 2024) – Large-scale free-text user classification	MLP, RBFN, SVM, RF	IKDD dataset	Introduced a large benchmark dataset for user classification; raw keystroke data was not publicly available due to privacy concerns.
2025 (Martins et al., 2025) – Intelligent biometric authentication using keystroke dynamics	KNN, RF, LGBM	KeyRecs dataset	LGBM achieved strong authentication performance with low error rates; model explainability was not explored.
2025 (Medvedev et al., 2025) – Decision-making framework for keystroke authentication	Siamese neural network, triplet loss	CMU and KeyRecs datasets	Improved detection of genuine and impostor users; training required higher computational resources.
2025 (Budzys et al., 2025) – Deep learning and data fusion for keystroke authentication	Siamese neural network, CNN, triplet loss	CMU, KeyRecs, GREYC-NISLAB	Combined multiple datasets to improve authentication robustness; increased model complexity.
2026 (Kaur et al., 2026) – Smartphone continuous authentication	CNN-LightGBM, LIME, SHAP	ExtraSensory dataset	Improved authentication accuracy and provided explainable decisions; evaluation was limited to smartphone sensor data.
2026 (Radosevich and Borowczak, 2026) – Continuous authentication subversion analysis	PCA, auto-sklearn, statistical forgery	University of Buffalo dataset	Showed that keystroke systems can be affected by synthetic typing attacks; no protection mechanism was proposed.

In this study, we explored the enhancement of traditional authentication methods by incorporating Keystroke Dynamics (KSD), which are based on user typing patterns. Deep learning and machine learning methods were applied to the keystroke data to identify sophisticated patterns derived from typing behavior. Our objective was to combine state-of-the-art feature engineering, sequential modeling, and ensemble methods to develop an authentication technique that is more secure, efficient, and scalable. Moreover, we prioritized the interpretability of the models to improve their transparency and trustworthiness.

## 3 Keystroke dynamics LSTM-ensemble model

**Figure 1:**
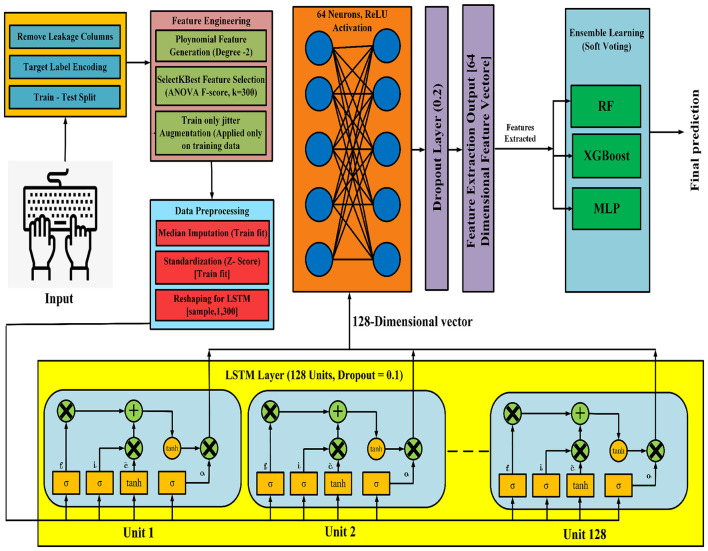
LSTM-ensemble keystroke authentication model (LEKAM).

The suggested architecture combines advanced feature engineering, LSTM-based temporal representation learning, and a hybrid ensemble classification framework to achieve user authentication using keystroke dynamics at a very high accuracy level. The first step in the process of typing data is to convert it into the final prediction through several processing layers, which allows for the extraction of both handcrafted and deep-learned representations. The architecture proposed in the current study is composed of four primary phases, which are depicted in detail in Figure 1. The phases are Feature Engineering and Preprocessing, Deep Temporal Representation Learning (LSTM Layer), and Hybrid Ensemble Classification (Soft Voting).

### 3.1 Keystroke dynamic datasets

Keystroke dynamics is a relatively unexplored biometric system that utilizes unique timing patterns and rhythms of individual typing styles. Keystroke dynamics has several advantages over other behavioral authentication methods, including low cost, non-invasiveness, transparency, and continuous monitoring. A high-quality keystroke dataset is crucial for determining the effectiveness of authentication methods based on keystroke dynamics (Shadman et al., 2025). Keystroke datasets vary in size, quality, and fixed or free text, which significantly affects the quality of the results. When keystrokes are captured in a natural typing manner while users are engaged in everyday computer use, they provide the most accurate measure of authentic typing behavior in the real world, allowing for an accurate and effective measure (Zhong et al., 2012).

These datasets are used by algorithms to examine time lags, or gaps, between key-presses and releases to make decisions about authentication. By examining the patterns of key-press and releases, along with the time lags between keystrokes, keystroke dynamics can reveal unique neurophysiological traits of individuals, such as handwriting or signatures, thus allowing for reliable and accurate identification of users (Patil and Renke, 2016). Some of the more commonly used datasets for keystroke dynamics are listed in Table 2 among which, the KDA dataset was utilized for this study (Killourhy and Maxion, 2009).

**Table 2:** Overview of existing keystroke dynamics datasets.

Dataset name	Participants	Text type	Demographics	Software used	Data size	Features
GREYC (Giot et al., 2009)	133	Fixed	Mixed (mainly academic users)	NA	7,555	22
Buffalow (Sun et al., 2016)	39	Free & fixed	Mixed	Keylogger	13,461	NA
H-MOG D (Sitová et al., 2016)	100	Free & fixed	Mixed (47F/53M)	NA	NA	60
KDA (Killourhy and Maxion, 2009)	57	Fixed	21F/30M, 8LH/43RH, Age 18–70	Windows application	20,401	34
Keystroke100 (Loy et al., 2007)	100	Fixed	N/A	Developed program	NA	NA
KeyRecs (Dias et al., 2023)	99	Free & Fixed	39F/60M, 8LH/91RH, Age 18–51, 20 Nationalities	Online platform	1.6M	60
Risto & Graven (Risto and Graven, 2024)	103	Fixed	Mostly university students	Keylogger	NA	50
KDC1 (Shenoy, 2024)	110	Fixed	Mixed	NA	1,240	54
EmoSury (Maalej and Kallel, 2020)	124	Free & fixed	Mixed	Dynamic web application	NA	14
Clarkson Univ. Keystroke (Monaco et al., 2013)	39	Free & fixed	Mixed	JavaScript Keylogger	10K	NA

Because this study used an existing dataset, user consent was obtained from the original data providers during data collection. To protect privacy, all personal or identifying details were removed before data were used. The dataset was handled according to data protection laws such as GDPR, with measures such as encryption and secure processing. After the study, the data were either safely deleted or stored in compliance with privacy regulations.

### 3.2 Feature engineering

This feature engineering process aims to transform raw keystroke data into meaningful features that capture each user's distinct typing behavior. Improving data quality is often more important for model performance than developing new algorithms. Therefore, feature engineering plays a crucial role, as well-designed features with strong predictive power can effectively address complex classification problems. Using domain knowledge, features can be engineered to enhance the effectiveness of learning algorithms (Rawat and Khemchandani, 2017; Verdonck et al., 2021). In the proposed framework, feature engineering consists of polynomial feature generation, feature selection, and behavioral jitter augmentation. These techniques are applied to enrich the feature space, reduce redundancy, and improve the robustness of the authentication model. The details of each technique are presented in the following subsections (Turner et al., 2011).

1. **Polynomial feature generation:** To capture complex nonlinear relationships among keystroke timing attributes, polynomial feature generation was performed using the *PolynomialFeatures* function from the Scikit-learn library with a polynomial degree of 2 and *include_bias=False*. Given an original feature vector *X* = [*x*_1_, *x*_2_, …, *x*_*n*_], the transformation generates squared terms and pairwise interaction features according to
  $$\Phi \left(X\right)=\left\{x_{i},x_{i}^{2},x_{i}x_{j}\right\}$$
  where *i, j* ∈ {1, 2, …, *n*} and *i* < *j*. This process enables the model to capture higher-order dependencies among typing characteristics. For example, interaction features such as *H*.*t*×*H*.*i* and *DD*.*period*.*t*×*UD*.*period*.*t* provide additional information about the relationships between hold times and latency measurements. In the DSL dataset, the original 31 timing features were expanded to 527 polynomial features, significantly enriching the feature space.
2. **Feature selection using SelectKBest:** Since polynomial expansion substantially increases the dimensionality of the feature space, feature selection was applied to remove redundant and less informative features. The *SelectKBest* algorithm with the ANOVA F-score criterion (*f_classif* ) was employed to evaluate the discriminative power of each feature with respect to the target user classes. The ANOVA F-score is computed as
  $$F=\frac{\text{Between-Class Variance}}{\text{Within-Class Variance}}$$
  where a larger F-score indicates a stronger relationship between the feature and the target class. Based on this criterion, the top 300 features were selected from the generated polynomial feature set. This dimensionality reduction step decreases computational complexity, improves model efficiency, and reduces the risk of overfitting while preserving the most informative typing characteristics.
3. **Behavioral jitter augmentation:** To improve model robustness and generalization, train-only jitter augmentation was applied to the training samples. Small Gaussian noise proportional to the standard deviation of each feature was added to the original timing values according to
  $$x_{jitter}=x+N\left(0,\alpha \sigma \right)$$
  where *x* is the original feature value, σ is the feature standard deviation, and α = 0.01 is the jitter factor used in this study. This augmentation strategy simulates natural variations in typing behavior caused by factors such as fatigue, stress, typing speed fluctuations, and hardware differences. Importantly, augmentation was applied only to the training set, while validation and testing data remained unchanged, thereby preventing information leakage and ensuring an unbiased evaluation of model performance.

Table 3 presents the feature engineering techniques employed in the proposed authentication framework. The original keystroke timing attributes, including hold times (H), key-down to key-down latencies (DD), and key-up to key-down latencies (UD), were first preprocessed and subsequently transformed through polynomial feature generation, feature selection, and data augmentation. Polynomial feature generation (degree = 2) was used to create interaction and squared terms that capture non-linear relationships among keystroke timing features. Subsequently, SelectKBest feature selection based on the ANOVA F-score was applied to retain the most discriminative features while reducing dimensionality. Finally, train-only jitter augmentation was performed by introducing small random perturbations to the training samples, thereby improving model generalization and robustness against natural typing variations.

**Table 3:** Engineered features used in the proposed model.

Feature name	Description	Formula/method	Category
H.t × H.i	Interaction between timing features	*H*.*t*·*H*.*i*	Interaction feature
DD.period.t × UD.period.t	Interaction between DD and UD timings	*DD*.*period*.*t*·*UD*.*period*.*t*	Interaction feature
H.e × UD.e.five	Interaction between hold-time and UD timing features	*H*.*e*·*UD*.*e*.*five*	Interaction feature
*c* ^2^	Squared term of feature *c*	*c* ^2^	Polynomial feature
*c*_*i*_×*c*_*j*_	Pairwise interaction between features	*c*_*i*_·*c*_*j*_	Polynomial feature
Top-300 features	Most informative features selected using ANOVA F-score	SelectKBest(*F*-score, *k* = 300)	Feature selection
c_jitter	Noise-enhanced feature for robustness	*c*+*N*(0, σ)	Data augmentation

**Figure 2:**
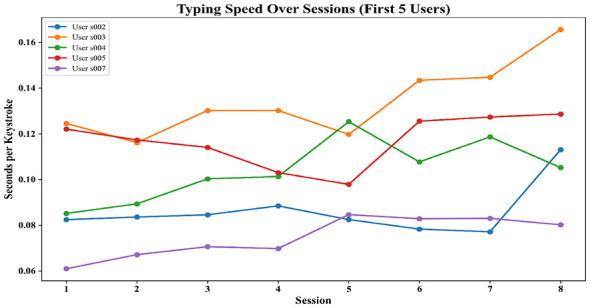
Session-wise typing speed patterns for sample users.

**Figure 3:**
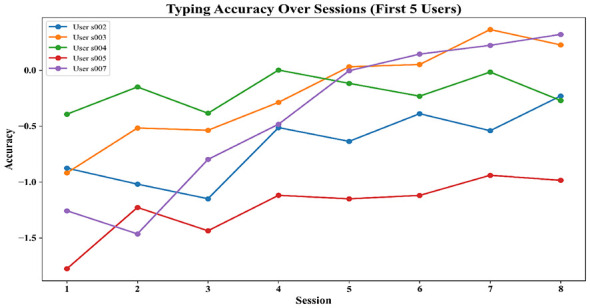
Session-wise typing performance patterns for sample users.

**Figure 4:**
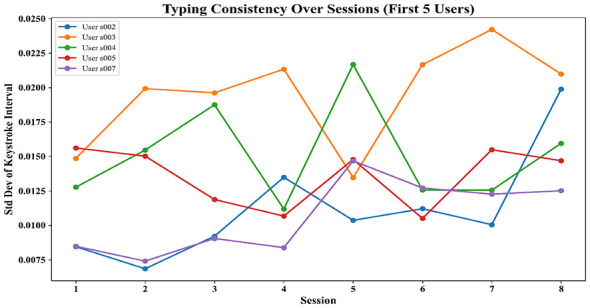
Session-wise typing consistency patterns for sample users.

Figures 2–4 present session-wise behavioral variations observed in the keystroke dynamics dataset. The results reveal user-specific differences in typing speed, performance, and temporal consistency across sessions. Such variations capture distinctive behavioral traits that can be effectively leveraged for user identification and authentication.

### 3.3 Pre-processing and visualization

It is important to pre-process and visualize keystroke datasets to maintain the data quality and unveil patterns. Pre-processing manages missing values, normalizes features, encodes data, and detects outliers to ensure consistency in the model (Maharana et al., 2022). Visualization approaches include heat maps, box plots, histograms, highlight distributions of features, correlations, and changes in user behaviors, making them clean, interpretable, and ready for robust modeling (Gumelar, 2019).

#### 3.3.1 Visualization

**Figure 5:**
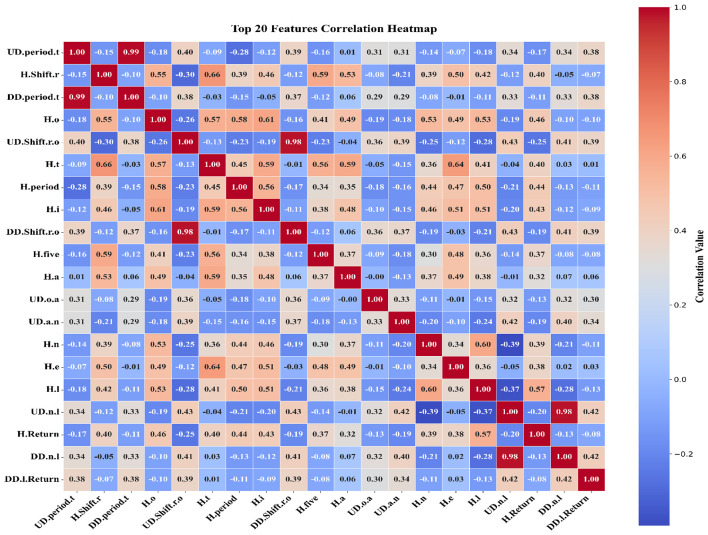
Correlation heatmap of the top 20 keystroke features.

The correlation heatmap provides an exploratory visualization of the relationships among the original keystroke timing features. Positive correlations indicate features that exhibit similar behavioral patterns, whereas negative correlations represent inverse relationships. The visualization helps reveal dependencies and interactions within the keystroke dynamics data, providing insights into user-specific typing behavior. These observations support the subsequent feature engineering and feature selection processes employed in the proposed framework (Kulkarni and Sinha, 2012). Figure 5 presents the correlation heatmap of the top 20 keystroke features.

**Figure 6:**
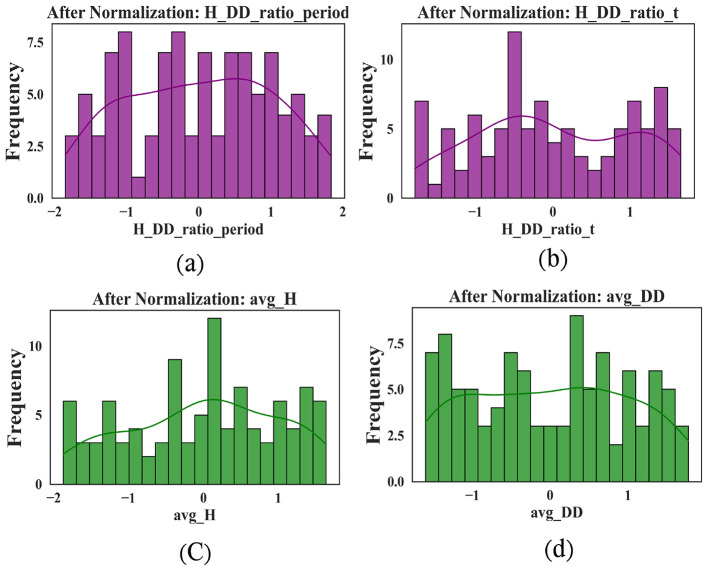
Normalized distributions of representative keystroke features. The distributions of **(a)** H_DD_ratio_period, **(b)** H_DD_ratio_t, **(c)** avg_H, and **(d)** avg_DD are shown after normalization.

Keystroke dynamics normalization prevents disparate features from dominating one another and creates a fair representation and comparison across models. In this study, we found that when we normalized the selected keystroke features, we observed relevant patterns, reduced the effects of outliers, and normalize appearance, and made the distributions easier and more comparative, as shown in Figure 6.

**Figure 7:**
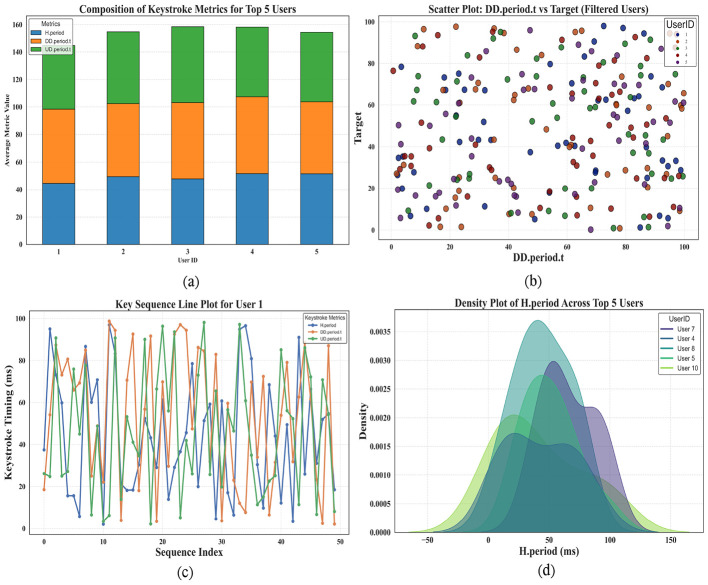
Visualization of keystroke dynamics characteristics. **(a)** User-wise metric composition, **(b)** DD.period.t vs. target-user scatter plot, **(c)** keystroke timing sequence for a sample user, and **(d)** H.period density distributions across users.

To comprehend and evaluate the latent patterns and correlations found in the data, it is necessary to visualize keystroke dynamics using different diagrams. The combination of keystroke metrics provides an overview of the impact of different timing features on user behavior, allowing the identification of dominant patterns and important features. A scatter plot showing two critical features enables us to evaluate the degree of correlation as well as the interactions between them, thus indicating that the various metrics are interconnected and could possibly have an impact on the correctness of authentication. The line graphs of the main sequences for the selected users not only reveal different typing styles but also demonstrate the impact of time on these habits, thus confirming that some people are the same over time while others change and have their keystroke patterns detected over a period of time. The graphical representations in Figure 7 provide help in the selection of features, the determination of the users' most important characteristics, and the facilitation of the creation of efficient user authentication and behavioral biometric solutions.

#### 3.3.2 Preprocessing

Before model training, several preprocessing steps were performed to prepare the keystroke dynamics dataset. The user identifier (*subject*) was used exclusively as the target label for the authentication task and was converted into numerical class labels using Label Encoding. To prevent data leakage, the session-related attributes (*sessionIndex*) and (*rep*) were removed from the input feature set, as these variables represent metadata rather than behavioral characteristics. All remaining keystroke timing attributes were converted to numeric format, and any invalid or missing values were handled using median imputation. Median imputation was selected because it is less sensitive to outliers and preserves the central tendency of the timing features more effectively than mean imputation.

Feature standardization was subsequently performed using StandardScaler, which was fitted only on the training data and then applied to the testing data to prevent information leakage. This process normalizes each feature to zero mean and unit variance, ensuring that features with different numerical ranges contribute equally during model training. After feature engineering and feature selection, the resulting feature matrix was reshaped into a three-dimensional tensor of shape (*N*, 1, *F*), where *N* denotes the number of samples and *F* represents the number of selected features. Each sample was represented as a single timestep containing the engineered keystroke attributes and their interaction features. This representation enables the LSTM network to learn discriminative behavioral patterns and extract user-specific feature representations for authentication.

### 3.4 Model development

Model creation is a step-by-step process that involves building, training, and evaluating a keystroke dynamics-based user authentication system. The steps below explain the complete workflow that was followed to develop the proposed LSTM-based ensemble classification model.

#### 3.4.1 Size of train, validation, and test splits

In this study, the dataset was initially divided into 80% training data and 20% testing data using stratified sampling to preserve the class distribution across all 51 users. The testing set was held out and used exclusively for final performance evaluation. To obtain a reliable assessment of model performance and stability, stratified 5-fold cross-validation was performed on the training set. During each fold, approximately 80% of the training subset was used for model training and the remaining 20% was used for validation while maintaining class balance. Consequently, each fold utilized approximately 64% of the entire dataset for training, 16% for validation, and 20% for testing. All preprocessing operations, including median imputation, polynomial feature generation, feature selection, and feature scaling, were fitted only on the training portion of each fold and subsequently applied to the corresponding validation portion to prevent information leakage. The final cross-validation performance was obtained by averaging the results across all five folds, providing a robust and unbiased evaluation of the proposed authentication framework.

#### 3.4.2 LSTM architecture

The LSTM model was employed to learn complex relationships among the engineered keystroke features for user authentication. The input layer receives data in the form of a three-dimensional tensor with the shape (*N*, 1, *F*), where *N* denotes the number of samples and *F* represents the selected feature set obtained after polynomial feature generation and feature selection. In the proposed implementation, the selected feature matrix containing 300 features was reshaped into a tensor of shape (*N*, 1, 300), where each sample is represented as a single timestep containing all engineered keystroke attributes. Although the input consists of a single timestep, the LSTM layer is capable of learning latent feature representations and complex nonlinear dependencies among the selected keystroke features. The extracted representations are subsequently used for user authentication. The detailed architecture of the model is presented in Table 4.

**Table 4:** LSTM model architecture summary.

Layer (type)	Output shape	Param #
InputLayer	(None, 1, 300)	0
LSTM (128 Units)	(None, 128)	219,648
Dense (ReLU, 64 Units)	(None, 64)	8,256
Dropout	(None, 64)	0
Dense (Softmax, 51 Units)	(None, 51)	3,315
Total params		231,219
Trainable params		231,219
Non-trainable params		0

##### 3.4.2.1 LSTM layer

The LSTM layer consists of 128 hidden units and is responsible for learning discriminative behavioral patterns from the engineered keystroke features. Although each sample is represented as a single timestep, the LSTM effectively models complex dependencies among the input features and generates a 128-dimensional feature representation that captures user-specific typing characteristics.

##### 3.4.2.2 Dense layer

The 128-dimensional output generated by the LSTM layer is passed to a fully connected dense layer containing 64 neurons with ReLU activation. This layer performs nonlinear feature transformation and dimensionality reduction, enabling the network to learn more compact and discriminative feature representations for user authentication.

##### 3.4.2.3 Dropout layer

To reduce the risk of overfitting and improve generalization performance, a dropout layer with a dropout rate of 0.2 is applied after the dense layer. During training, 20% of the neurons are randomly deactivated in each iteration, preventing excessive reliance on specific neurons and encouraging the network to learn more robust feature representations.

##### 3.4.2.4 Output layer

The output layer consists of a fully connected layer with one neuron for each user class. A Softmax activation function is applied to convert the output scores into a probability distribution over all users. The user corresponding to the highest probability is selected as the final authentication result.

### 3.5 Ensemble classifiers

The LSTM model generates a 64-dimensional feature vector from the dropout layer, which is then fed into an ensemble classifier. The ensemble combines predictions from different machine learning models to improve accuracy and reliability and containes three different machine learning models: Random Forest (Kulkarni and Sinha, 2012), XGBoost (Chen and Guestrin, 2016), and MLPClassifier (Rashedi et al., 2024). The Random Forest is configured with n_estimators = 100, which ensures that the model builds 100 decision trees to boost the robustness. To properly address the multiclass classification problem, the XGBoost classifier is configured with n_estimators = 100 and eval_metric=“mlogloss,” as well as label encoding disabled with use_label_encoder = False. The MLPClassifier has a single hidden layer of 64 neurons [hidden_layer_sizes = (64,)], and is trained for up to 500 iterations (max_iter). The soft voting technique aggregates the predicted probabilities from all three classifiers before selecting the class with the highest average probability as the final output (Mienye and Sun, 2022).

## 4 Experimental analysis

### 4.1 Experimental setup and dataset details

The experiments were conducted using a Jupyter Notebook on a local machine with an Intel Core i7 (8th Gen) CPU, an NVIDIA Quadro P5000 GPU, and 16GB RAM. The programming environment included Python 3.12 and the main libraries were Scikit-learn, Pandas, NumPy, Seaborn, and Matplotlib for machine learning, data handling, and visualization. Reliable results were assured by applying preprocessing methods and evaluation metrics such as accuracy and ROC-AUC. A memory profiler was employed to monitor resource consumption and enhance performance. A KDA keystroke dynamics dataset was used in this study (Killourhy and Maxion, 2009).

### 4.2 Evaluation metrics

We evaluated the performance of the model using a range of metrics including accuracy, specificity, sensitivity, Precision, F1-Score, False Negative Rate (FNR), False Positive Rate (FPR), Negative Predictive Value (NPV), Matthews Correlation Coefficient (MCC), and Equal Error Rate (EER). Training Time was measured to determine the efficiency of the model (Miller et al., 2024).

1. **Accuracy:** Percentage of correctly classified instances from all examples. $\text{Accuracy}=\frac{\text{TP}+\text{TN}}{\text{TP}+\text{TN}+\text{FP}+\text{FN}}$
2. **Precision:** Percentage of correct positive predictions from all positive predictions made by the model. $\text{Precision}=\frac{\text{TP}}{\text{TP}+\text{FP}}$
3. **Specificity:** Evaluates the model's ability to correctly identify negative cases and reject negatives. $\text{Specificity}=\frac{\text{TN}}{\text{TN}+\text{FP}}$
4. **Sensitivity:** This evaluates the model's ability to correctly identify positive cases. $\text{Sensitivity}=\frac{\text{TP}}{\text{TP}+\text{FN}}$
5. **F1-score:** The F1-score combines Precision and Sensitivity and balances them to provide a more accurate metric, particularly when there are more false positives or negatives. $\text{F1}=\frac{2\times \text{Precision}\times \text{Sensitivity}}{\text{Precision}+\text{Sensitivity}}$
6. **Matthews correlation coefficient (MCC):** A balanced binary classification metric that considers all four confusion matrix terms. $\text{MCC}=\frac{\left(\text{TP}\cdot \text{TN}\right)-\left(\text{FP}\cdot \text{FN}\right)}{\sqrt{\left(\text{TP}+\text{FP}\right)\left(\text{TP}+\text{FN}\right)\left(\text{TN}+\text{FP}\right)\left(\text{TN}+\text{FN}\right)}}$
7. **Equal error rate (EER):** Defines the point at which the False Acceptance Rate (FAR) and FRR occur. It is commonly calculated by using the ROC curve at the threshold, where FAR = FRR.

### 4.3 Experimental details

The performance of the developed authentication framework was evaluated using several classification metrics, including Accuracy, Recall, Precision, F1-Score, Matthews Correlation Coefficient (MCC), Equal Error Rate (EER), and Training Time (TT). Table 5 presents the comparative performance of the proposed model against various deep learning, transformer-based, and ensemble-based approaches.

**Table 5:** Performance comparison across models.

Model	Acc	Recall	Prec	F1 score	MCC	EER	TT (sec)
CNN + Soft voting (LGB+CAT+XGB)	62.70	62.70	62.40	62.16	61.97	0.0780	706.98
LSTM + RF	89.46	89.46	89.58	89.44	89.25	0.0229	118.67
LSTM + Stacking (MLP+LGB) Meta = LR	89.85	89.85	90.08	89.89	89.65	0.0336	276.51
Transformer	89.93	89.93	90.11	89.95	89.73	0.0182	239.81
Transformer + Stacking (LGB+CAT+XGB) Meta = XGB	90.29	90.29	90.41	90.28	90.10	0.0196	1,260.02
Transformer + Soft Voting (LGB+CAT+XGB)	90.54	90.54	90.67	90.54	90.35	0.0181	337.17
Transformer + Stacking (LGB+CAT+XGB) Meta = LR	90.59	90.59	90.81	90.63	90.40	0.0281	1,121.42
Stacking (LGB+CAT+XGB) Meta = LR	93.90	93.90	94.10	93.92	93.78	0.0175	3,234.57
Stacking (LGB+CAT+XGB) Meta = XGB	93.92	93.92	94.04	93.91	93.80	0.0101	3,652.05
Proposed model	94.75	94.89	94.75	94.76	94.65	0.0100	188.10

Acc, Accuracy; Prec, Precision; TT, Training Time (sec).

**Figure 8:**
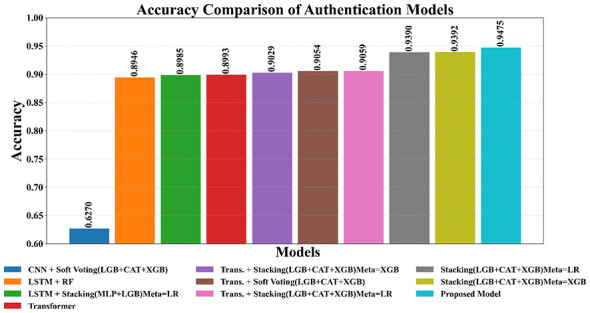
Accuracy comparison.

Accuracy measures the overall proportion of correctly classified authentication instances. Among the evaluated models, the CNN + Soft Voting (LGB+CAT+XGB) model achieved the lowest accuracy of 62.70%, indicating limited suitability for keystroke-based authentication. The LSTM + RF and LSTM + Stacking (MLP+LGB) Meta = LR models achieved accuracies of 89.46% and 89.85%, respectively. Transformer-based approaches demonstrated improved performance, with accuracies ranging from 89.93% to 90.59%. The ensemble-based Stacking (LGB+CAT+XGB) models achieved accuracies of 93.90% and 93.92%. The proposed model achieved the highest accuracy of 94.75%, demonstrating superior user authentication performance. Figure 8 presents the comparative accuracy achieved by all evaluated models.

Recall measures the ability of a model to correctly identify genuine users. The proposed model achieved the highest recall of 94.89%, outperforming all competing approaches. The transformer-based and ensemble-based models achieved recall values between 89.93% and 93.92%, while the LSTM + RF model achieved 89.46%. The high recall of the proposed framework indicates its effectiveness in minimizing false rejections of legitimate users.

Precision evaluates the correctness of positive authentication decisions. The proposed model achieved a precision of 94.75%, which is comparable to the best-performing ensemble models and higher than the transformer-based and LSTM-based approaches. The high precision value indicates that the proposed framework effectively reduces the likelihood of incorrectly accepting impostors as legitimate users.

The F1-Score provides a balanced assessment of precision and recall. The proposed model achieved the highest F1-Score of 94.76%, followed by the Stacking (LGB+CAT+XGB) Meta = LR and Meta = XGB models, which achieved F1-Scores of 93.92% and 93.91%, respectively. These results demonstrate the ability of the proposed framework to maintain a strong balance between user acceptance and rejection decisions.

MCC is a robust performance metric that considers true and false predictions across all classes. The proposed model achieved the highest MCC value of 94.65%, indicating strong and balanced classification performance. In comparison, the best ensemble-based baseline achieved an MCC of 93.80%, while transformer-based models achieved MCC values between 89.73% and 90.40%.

**Figure 9:**
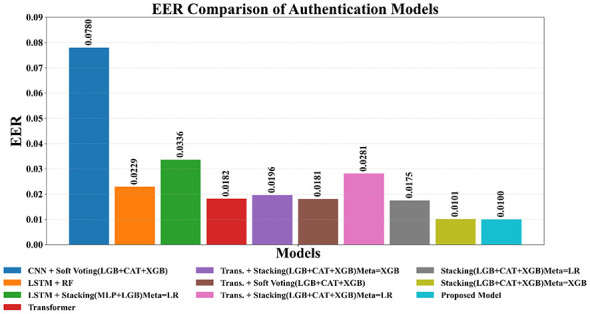
EER comparison.

The Equal Error Rate (EER) represents the operating point at which the False Acceptance Rate (FAR) equals the False Rejection Rate (FRR). Lower EER values indicate better authentication performance. The proposed model achieved an EER of 0.0100, which is among the lowest values obtained in the study and is comparable to the Stacking (LGB+CAT+XGB) Meta = XGB model (0.0101). Both models significantly outperformed the LSTM-based and transformer-based baselines, confirming their effectiveness in minimizing authentication errors. Figure 9 illustrates the EER comparison among the evaluated models.

**Figure 10:**
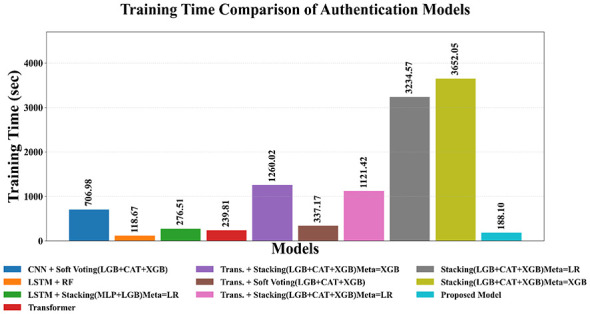
Training time comparison.

Training time is an important factor for practical deployment. The proposed model required 188.10 seconds for training, which is substantially lower than the computationally expensive stacking-based models, including Stacking (LGB+CAT+XGB) Meta = LR (3,234.57 s) and Stacking (LGB+CAT+XGB) Meta = XGB (3,652.05 s). Although the LSTM + RF model required a shorter training time of 118.67 seconds, its authentication performance was considerably lower than that of the proposed model. Therefore, the proposed framework provides an effective trade-off between computational efficiency and authentication accuracy. Figure 10 shows the training time comparison of all evaluated models. Overall, the proposed hybrid authentication framework achieved the best combination of Accuracy, Recall, Precision, F1-Score, MCC, EER, and training efficiency. These results demonstrate that the integration of LSTM-based feature learning with ensemble classification provides a reliable and effective solution for keystroke dynamics-based user authentication.

### 4.4 LSTM model performance and results

**Figure 11:**
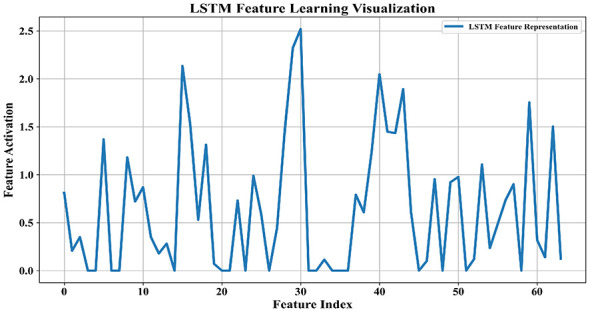
LSTM feature learning visualization.

The LSTM layer was employed to learn discriminative feature representations from the engineered keystroke features. Although each sample was represented as a single timestep containing the selected keystroke attributes, the LSTM effectively captured nonlinear relationships among the input features and generated a compact 128-dimensional representation of each user's typing behavior. This feature vector serves as a high-level embedding that summarizes user-specific characteristics and facilitates subsequent classification. The LSTM Feature Learning Visualization shown in Figure 11 presents the extracted feature activations obtained from the first training sample. The observed variations in feature values indicate that the LSTM learned diverse latent representations from the keystroke dynamics data. These learned features provide a compact description of user-specific typing patterns and improve the separability of different users during classification.

**Figure 12:**
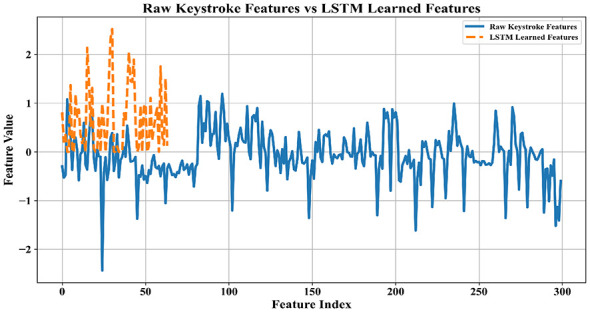
Raw data vs. LSTM output.

**Figure 13:**
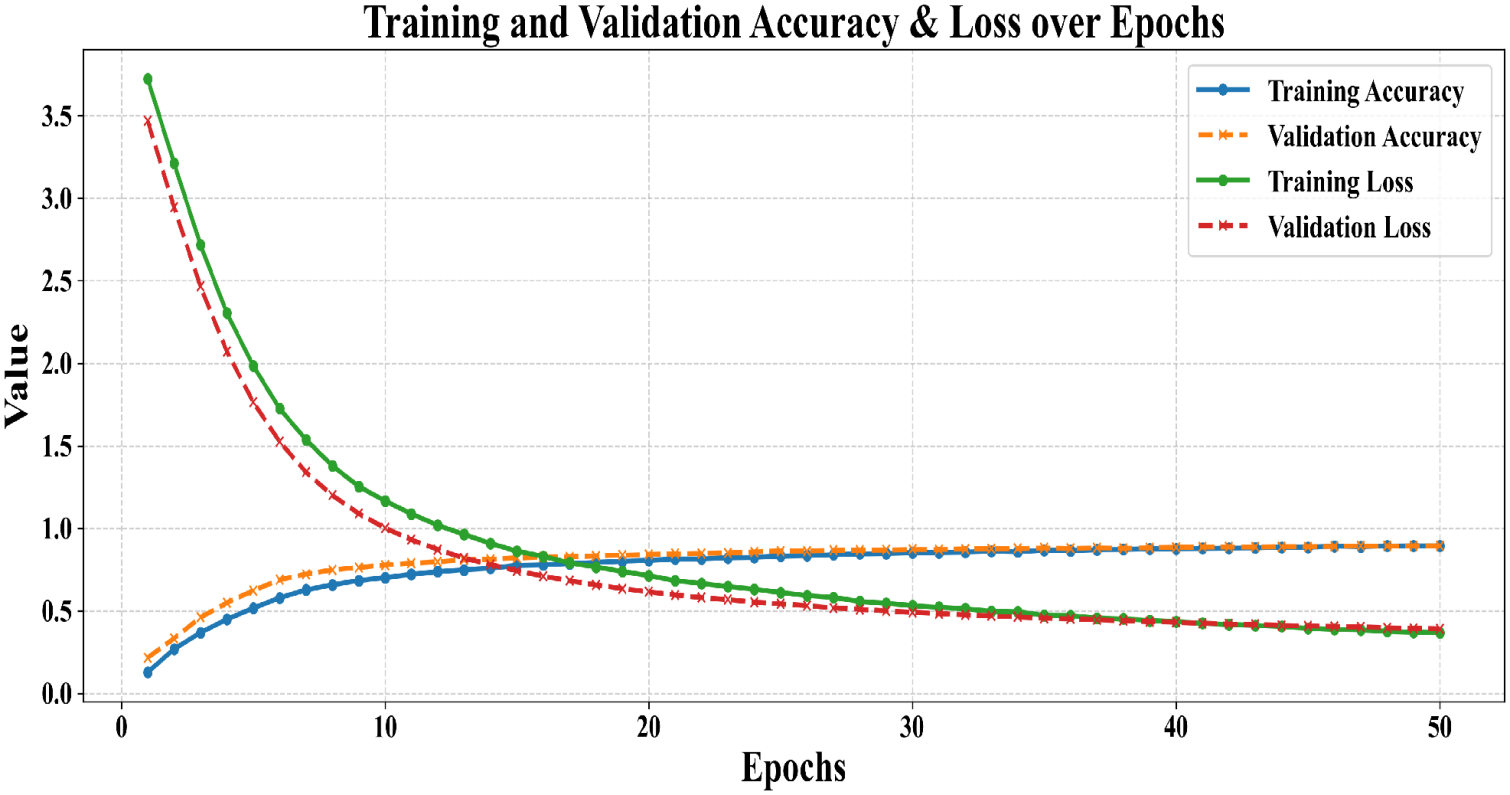
LSTM training and validation accuracy and loss curve.

**Figure 14:**
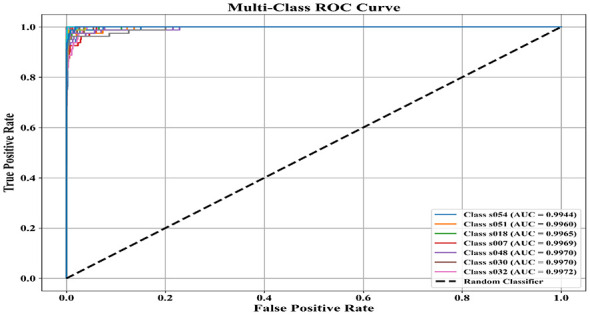
ROC curve.

The relationship between the input keystroke features and the corresponding LSTM-generated feature representation is illustrated in Figure 12. The figure demonstrates the ability of the LSTM layer to transform the original keystroke attributes into a more informative feature space, thereby enhancing the discriminative capability of the authentication framework. Figure 13 presents the training and validation accuracy and loss curves obtained during model training. The convergence behavior indicates stable learning with minimal overfitting, demonstrating the effectiveness of the proposed architecture. To further evaluate the classification performance, a One-vs.-Rest (OvR) multiclass ROC analysis was performed. Since the authentication framework classifies 51 users, a separate ROC curve was generated for each class. Figure 14 illustrates the ROC curves and corresponding Area Under the Curve (AUC) values, demonstrating the ability of the proposed framework to distinguish between users with different typing patterns.

### 4.5 Computational complexity and model inference efficiency analysis

**Figure 15:**
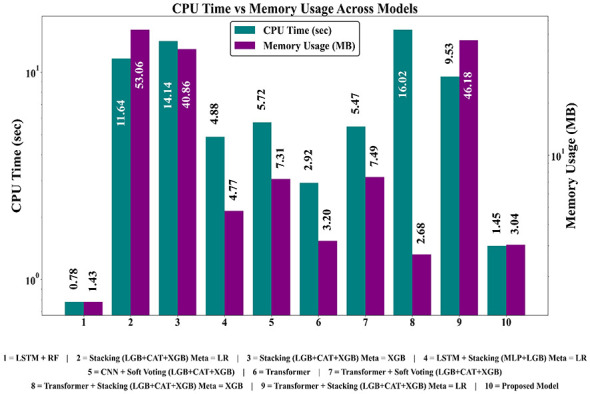
CPU time and memory usage comparison across models.

A comparison of the proposed model with other authentication models in terms of real-time inference efficiency and computational resource utilization is presented in Table 6. The results demonstrate that the proposed model achieves an effective balance between authentication performance and computational efficiency. The CPU time and memory consumption of the evaluated models are illustrated in Figure 15. The proposed model required only 1.4531 seconds of CPU time and 3.0391 MB of memory during inference, which is substantially lower than computationally intensive models such as Transformer + Stacking (LGB+CAT+XGB) Meta = XGB, which required 16.0156 seconds of CPU time, and Stacking (LGB+CAT+XGB) Meta = LR, which required 11.6406 seconds of CPU time and 53.0625 MB of memory. Although the LSTM + RF model achieved the lowest inference time and memory consumption, its authentication performance was considerably lower than that of the proposed model.

**Table 6:** Inference efficiency comparison of proposed vs. existing models.

Model	Inference			Throughput (Samples/s)	Detection time (ms/Sample)	Reaction time (ms)
	Wall (sec)	CPU (sec)	Memory (MB)			
CNN + soft voting (LGB+CAT+XGB)	1.2684	5.7188	7.3125	3,216.55	0.3109	10.3109
LSTM + RF	0.2323	0.7812	1.4258	17,563.17	0.0569	10.0569
LSTM + stacking (MLP+LGB) Meta = LR	1.1383	4.8750	4.7734	3,584.34	0.2790	10.2790
Transformer	1.9148	2.9219	3.2031	2,130.73	0.4693	10.4693
Transformer + stacking (LGB+CAT+XGB) Meta = XGB	3.3365	16.0156	2.6797	1,222.83	0.8178	10.8178
Transformer + soft voting (LGB+CAT+XGB)	1.2288	5.4688	7.4922	3,320.28	0.3012	10.3012
Transformer + stacking (LGB+CAT+XGB) Meta = LR	2.3412	9.5312	46.1797	1,742.72	0.5738	10.5738
Stacking (LGB+CAT+XGB) Meta = LR	2.6363	11.6406	53.0625	1,547.64	0.6461	10.6461
Stacking (LGB+CAT+XGB) Meta = XGB	2.8356	14.1406	40.8633	1,438.87	0.6950	10.6950
Proposed model	0.2535	1.4531	3.0391	16,092.93	0.0621	10.0621

In terms of throughput, the proposed model processed 16,092.93 samples per second, which is significantly higher than most transformer-based and stacking-based approaches. The proposed model achieved a detection time of 0.0621 ms/sample, which is comparable to the LSTM + RF model (0.0569 ms/sample) and substantially lower than Transformer + Stacking (LGB+CAT+XGB) Meta = XGB (0.8178 ms/sample). Furthermore, the proposed model achieved a reaction time of 10.0621 ms, which is among the lowest values observed and comparable to the fastest competing models. These results indicate that the proposed framework is capable of delivering high authentication accuracy while maintaining low computational overhead and fast response times, making it suitable for real-time keystroke dynamics-based user authentication systems.

### 4.6 Statistical analysis

Table 7 presents the results of the k-fold cross-validation analysis performed on the proposed authentication framework. The model achieved a mean accuracy of 93.99% with a standard deviation of ±0.26% under 5-fold cross-validation, indicating consistent performance across different data partitions. Under 10-fold cross-validation, the model achieved a slightly higher mean accuracy of 94.08% with a standard deviation of ±0.72%. The corresponding 95% confidence intervals were [93.67, 94.31] and [93.57, 94.60] for the 5-fold and 10-fold evaluations, respectively. The relatively narrow confidence intervals and low standard deviation values demonstrate the stability, robustness, and generalization capability of the proposed model across different validation folds. Overall, the results confirm that the proposed framework provides reliable and consistent authentication performance (Wong, 2015).

**Table 7:** Assessing the robustness of the proposed model using K-fold cross-validation.

K-fold validation	Mean accuracy (%)	Std. deviation (±%)	95% confidence interval
5-Fold	93.99	±0.26	[93.67, 94.31]
10-Fold	94.08	±0.72	[93.57, 94.60]

### 4.7 Ablation study

To assess the contribution of individual components, an ablation study was conducted by evaluating different configurations of the proposed authentication framework. As shown in Table 8, the Raw Features + Voting model achieved an accuracy of 89.71%, while Polynomial Features + Voting improved the accuracy to 93.82%, indicating the importance of feature engineering. The single-classifier LSTM variants achieved accuracies between 89.19% and 90.07%, demonstrating that the LSTM feature extractor effectively captures user-specific typing patterns.

**Table 8:** Ablation study of the proposed authentication framework.

Configuration	Accuracy (%)	Time (Sec)
Raw features + voting	89.71	54.40
Polynomial features + voting	93.82	686.66
LSTM + RF	89.46	101.20
LSTM + XGB	89.19	141.55
LSTM + MLP	90.07	154.91
LSTM + RF + XGB (Without MLP)	89.75	202.30
LSTM + RF + MLP (Without XGB)	89.58	149.83
LSTM + XGB + MLP (Without RF)	90.49	209.23
Full model (100 features)	89.71	–
Full model (200 features)	93.50	–
Full proposed model (300 features)	94.75	226.05

Further analysis was performed by removing one classifier at a time from the ensemble. The reduced ensemble configurations achieved accuracies ranging from 89.58% to 90.49%, confirming that each classifier contributes useful complementary information. The Full Proposed Model, which combines LSTM-based feature learning with RF, XGB, and MLP soft voting, achieved the highest accuracy of 94.75%. These results demonstrate that the integration of LSTM feature extraction and ensemble classification provides the most effective and reliable authentication performance.

### 4.8 Adversarial attack resistance analysis

To evaluate the robustness of the proposed authentication framework against adversarial attacks, four widely used attack methods were considered: Fast Gradient Sign Method (FGSM), Projected Gradient Descent (PGD), Basic Iterative Method (BIM), and Carlini-Wagner L2 (CW-L2) (Costa et al., 2024). In this study, adversarial perturbations were generated using only the LSTM feature-learning component of the framework, since gradient-based attacks require access to differentiable model parameters. The generated adversarial samples were subsequently evaluated using the complete authentication pipeline consisting of the LSTM feature extractor and the stacking classifier.

Table 9 presents the attack resistance results. The original authentication accuracy of the proposed framework was 94.75%. As the perturbation magnitude (ε) increased, a gradual reduction in accuracy was observed for all attack types. Under FGSM attacks, the accuracy decreased from 88.21% at ε = 0.005 to 73.31% at ε = 0.05, resulting in a maximum failure rate of 21.44%. Similarly, BIM reduced the accuracy from 88.24% to 72.57%, corresponding to a failure rate of 22.18% at the highest perturbation level. Among the gradient-based attacks, PGD demonstrated the strongest robustness, maintaining an accuracy of 78.24% at ε = 0.05 with a failure rate of 16.51%. The CW-L2 attack produced the largest performance degradation, reducing the accuracy from 94.75% to 68.98% and yielding a failure rate of 25.77%.

**Table 9:** Adversarial attack resistance analysis of the proposed authentication framework.

Attack type	ε	Original acc. (%)	Post-attack acc. (%)	Failure rate (%)
FGSM	0.005	94.75	88.21	6.54
	0.01	94.75	87.25	7.50
	0.02	94.75	84.49	10.26
	0.05	94.75	73.31	21.44
PGD	0.005	94.75	88.38	6.37
	0.01	94.75	87.67	7.08
	0.02	94.75	86.18	8.57
	0.05	94.75	78.24	16.51
BIM	0.005	94.75	88.24	6.51
	0.01	94.75	87.21	7.54
	0.02	94.75	84.41	10.34
	0.05	94.75	72.57	22.18
CW-L2	L2 Norm	94.75	68.98	25.77

Overall, the proposed framework maintained relatively high authentication accuracy under low and moderate adversarial perturbations and demonstrated resilience against multiple attack strategies. Although performance degradation was observed as the attack strength increased, the framework retained substantial discriminative capability even under strong adversarial conditions. These results indicate that the learned keystroke representations remain robust and support reliable user authentication in the presence of adversarially manipulated inputs.

### 4.9 Evaluation on benchmark datasets and comparison with recent studies

The proposed authentication framework was evaluated using three publicly available keystroke dynamics datasets, namely KDA, KDC1 (Shenoy, 2024), and KeyRecs (Dias et al., 2023). Accuracy was used as the primary evaluation metric to assess its performance across different datasets. The model achieved accuracies of 94.75%, 93.55%, and 94.12% on the KDA, KDC1, and KeyRecs datasets, respectively, demonstrating consistent performance across diverse keystroke environments.

To further assess its effectiveness, the obtained results were compared with several recent keystroke authentication studies, as presented in Table 10. The compared studies reported accuracy values ranging from 81.16% to 98.7% and EER values ranging from 0.20% to 13.281%. However, direct comparison of accuracy values should be interpreted carefully because some studies focused on different application scenarios, such as smartphone-based authentication and keystroke attack analysis. In contrast, the proposed approach focuses on keystroke dynamics authentication and integrates polynomial feature generation, feature selection, jitter augmentation, LSTM-based temporal learning, ensemble classification, SHAP-based explainability, and adversarial attack evaluation within a unified framework. The model achieved an accuracy of 94.75%, an EER of 1.00%, and a detection time of 0.0621 ms/sample. In addition, it provides model interpretability, evaluates robustness against adversarial attacks, and supports efficient real-time authentication. Overall, these results demonstrate that the proposed approach offers a balanced combination of authentication performance, robustness, interpretability, and computational efficiency across different keystroke dynamics datasets.

**Table 10:** Technical comparison of the proposed framework with recent keystroke dynamics authentication studies.

Studies	Dataset/validation	Main technique	XAI/attack	Performance/efficiency
Yang et al. (2024)	Dhakal; 10 runs	Timing, soft-biometric, editing; FedAvg + CNN	×/ ×	Acc. 84.36%; Time N/A
Tsimperidis et al. (2024)	IKDD; 10-fold CV	Timing features, outlier filtering; RBFN/SVM	×/ ×	Acc. 81.2%; Time 0.96–1.14 s
Martins et al. (2025)	KeyRecs; 70:30 + 5-fold CV	Dwell/flight features; LGBM	×/ ×	Acc. 81.16%; EER 0.20%
Medvedev et al. (2025)	CMU + KeyRecs; user-wise split	GAFMAT, interpolation; SNN + triplet loss	×/ ×	Acc. 89.5%; EER 10.5%
Budzys et al. (2025)	CMU + KeyRecs + GREYC; 70:15:15	Data fusion, GAFMAT; SNN + triplet loss	×/ ×	EER 13.281%
Kaur et al. (2026)	ExtraSensory; nested CV	Sensor features, PCA; CNN-LightGBM	✓ ×	Acc. 98.7%; EER 2.07%; Inference 0.05 s
Radosevich and Borowczak (2026)	UB Dataset; session split	PCA, Auto-Sklearn; synthetic attack	×/✓	Acc. 98.7%; Time N/A
Proposed method	DSL/KDA; 80:20 + 5-fold CV	Polynomial features, feature selection, jitter augmentation; LSTM-based ensemble (RF, XGB, MLP)	✓✓	Acc. 94.75%; EER 1.00%; detection 0.0621 ms/sample

## 5 Model explainability using XAI

Artificial intelligence (AI) harnesses computers and technology to replicate human reasoning and address real-world challenges. With AI employing machine learning (ML) and deep learning, it predicts accurately, without human involvement. However, the complexity of the models makes them difficult to interpret. XAI addresses this challenge by providing understandable justifications for AI's decisions, producing assurances of transparency, confidence, and accountability in many industries, including healthcare, finance, and cybersecurity (Mokoena and Sabatta, 2020).

In this study, we utilized SHapley Additive exPlanations (SHAP) (Costa et al., 2024), an XAI method for revealing the inner workings of ML models, by presenting the contributions of each feature to the prediction. SHAP offers global interpretability and local interpretability at the same time; through the global interpretability app, the feature relevance is highlighted across the dataset in an aggregate sense, and the local interpretability app explains one prediction using force, decision, and waterfall plots. Utilizing Shapley values from game theory, SHAP provide a justification that is fair, consistent, and intuitively helpful, thus making it a trustworthy tool for comprehending the model's behavior. Moreover, to increase the model's explanation and use its real-world implications, SHAP was used to determine the impact of keystroke features such as hold time, keystroke latency, and typing rhythm on authentication decision-making. Therefore, SHAP assists us into pinpoint the specific features within the dataset that are the most influential in the model's prediction. This knowledge is particularly important in decision-making situations in practice across various industries such as cybersecurity, healthcare, and finance which rely on model-driven decisions that need to be justifiable to gain trust or meet regulatory requirements.

**Figure 16:**
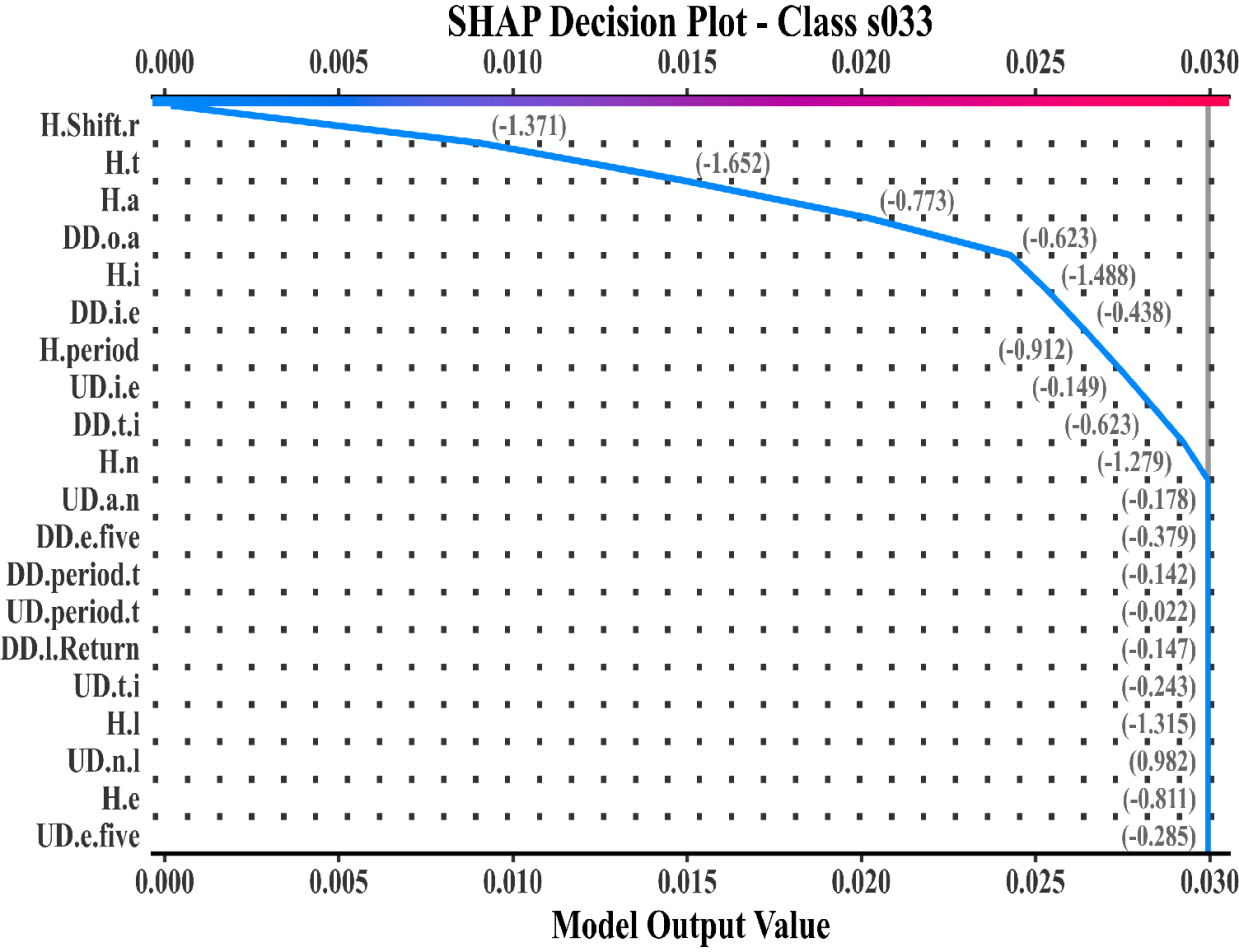
Decision plot.

Moreover, SHAP techniques point out the regions within the model that might be biased, and help us to diagnose such biases, especially those based on demographics such as typing speed and age, thus promoting fairness and equity. Visualizations such as decision plots and summary plots are the main ways in which stakeholders can pinpoint the model's route to decisions, regardless of whether they are non-technical or technical. In other word, these visualizations open the door to the model's behavior, making it transparent and articulating to users, thus enhancing its reliability and trustworthiness. The SHAP Decision Plot shown in Figure 16 illustrates how individual keystroke features contribute to the prediction of class s033. The horizontal axis represents the model output value, while the feature values for the selected sample are displayed in parentheses. The prediction path is influenced by several keystroke timing features, including H.Shift.r, H.t, H.a, DD.o.a, H.i, DD.i.e, H.period, and UD.i.e. Among these features, H.Shift.r, H.t, and H.a exhibit relatively larger contributions to the model output, indicating that hold-time and digraph timing characteristics play an important role in identifying the user. Features such as DD.t.i, H.n, UD.a.n, DD.e.five, DD.period.t, and UD.period.t contribute smaller adjustments to the prediction score. The cumulative effect of these feature contributions determines the final model output for the selected class. Overall, the decision plot demonstrates that the authentication decision is influenced by a combination of multiple keystroke timing features rather than a single dominant attribute, highlighting the ability of the proposed framework to capture complex behavioral typing patterns for user authentication.

**Figure 17:**
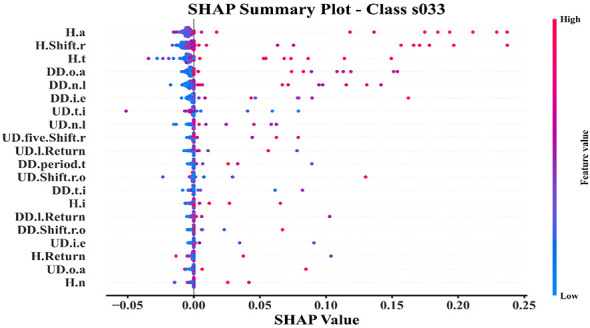
Summary plot.

The SHAP Summary Plot shown in Figure 17 presents the global importance of the keystroke features for predicting class s033. Each point represents an individual sample, where the horizontal position corresponds to the SHAP value and the color indicates the feature value, ranging from low (blue) to high (red). Features are ranked according to their overall impact on the model output. Among the evaluated features, H.a, H.Shift.r, H.t, DD.o.a, DD.n.l, and DD.i.e exhibited the largest SHAP value distributions, indicating that they contributed most significantly to the classification decisions. Positive SHAP values increased the likelihood of assigning a sample to class s033, whereas negative SHAP values reduced this likelihood. High feature values (red points) generally corresponded to larger positive SHAP contributions, while low feature values (blue points) tended to have smaller or negative effects on the prediction.

Most samples were concentrated around a SHAP value of zero, indicating limited influence for many observations, whereas a smaller number of samples showed large positive contributions reaching approximately 0.25. This distribution demonstrates that the model primarily relies on a subset of highly informative keystroke timing features, including hold-time, dwell-time, and flight-time characteristics. Overall, the SHAP summary plot confirms that multiple keystroke dynamics features jointly contribute to the authentication decision and provides insight into the global behavior of the proposed model.

## 6 Conclusion

Conventional authentication methods such as passwords and PINs are becoming increasingly vulnerable, motivating the adoption of behavioral biometric techniques such as Keystroke Dynamics (KSD) (Shenoy, 2024). KSD utilizes the unique typing characteristics of individuals for user authentication and identity verification. This study proposed an LSTM-based ensemble framework for keystroke dynamics authentication. The Long Short-Term Memory (LSTM) network was employed to capture latent relationships among keystroke timing features and learn user-specific behavioral patterns. The extracted feature representations were subsequently classified using an ensemble of Random Forest (RF), XGBoost (XGB), and Multilayer Perceptron (MLP) classifiers integrated through a soft-voting mechanism. The proposed framework also incorporated feature engineering techniques to enhance the discriminative capability of the keystroke data and improve authentication performance.

The proposed framework was evaluated using the KDA Keystroke Dynamics dataset. Experimental results demonstrated strong authentication performance, achieving an accuracy of 94.75%, recall of 94.89%, precision of 94.75%, F1-score of 94.76%, and MCC of 94.65%. The model also achieved a low Equal Error Rate (EER) of 0.0100, indicating a balanced trade-off between false acceptance and false rejection rates. In terms of computational efficiency, the proposed framework achieved a throughput of 16,092.93 samples per second and a detection time of only 0.0621 ms per sample, making it suitable for real-time authentication applications. Furthermore, the model required only 3.0391 MB of memory during inference, with a wall-clock inference time of 0.2535 seconds, demonstrating its resource efficiency and scalability for practical deployment.

Moreover, statistical validation using k-fold cross-validation confirmed the stability and generalization capability of the proposed framework across different data partitions. Adversarial robustness analysis using FGSM, PGD, BIM, and CW-L2 attacks demonstrated that the model maintained effective authentication performance under various attack scenarios. In addition, Explainable Artificial Intelligence (XAI) techniques, particularly SHAP, were employed to improve model transparency and provide insights into the contribution of individual keystroke features toward authentication decisions. Future work will focus on incorporating contextual and behavioral factors, such as emotional state and cognitive variations during typing, to further improve the robustness and adaptability of keystroke dynamics-based authentication systems.

## Data Availability

Publicly available datasets were analyzed in this study. This data can be found here: https://www.kaggle.com/datasets/carnegiecylab/keystroke-dynamics-benchmark-data-set.

## References

[B1] Admass W. S. Munaye Y. Y. Diro A. A. (2023). Cyber security: state of the art, challenges and future directions. Cyber Secur. Applic. 2:100031. doi: 10.1016/j.csa.2023.100031

[B2] Al-Obaidi N. M. Al-Jarrah M. M. (2016). “Statistical median-based classifier model for keystroke dynamics on mobile devices,” in Proceedings of the sixth international conference on digital information processing and communications, Beirut, Lebanon, 186–191. doi: 10.1109/ICDIPC.2016.7470816

[B3] Altwaijry N. (2020). Keystroke dynamics analysis for user authentication using a deep learning approach. Int. J. Comput. Sci. Netw. Secur. 20, 209–216.

[B4] Altwaijry N. (2023). Authentication by keystroke dynamics: the influence of typing language. Appl. Sci. 13:11478. doi: 10.3390/app132011478

[B5] Ayotte B. Banavar M. Hou D. Schuckers S. (2020). Fast free-text authentication via instance-based keystroke dynamics. IEEE Trans. Biometr. Behav. Identity Sci. 2, 377–387. doi: 10.1109/TBIOM.2020.3003988

[B6] Bhana B. Flowerday S. (2020). Passphrase and keystroke dynamics authentication: usable security. Comput. Secur. 96:101925. doi: 10.1016/j.cose.2020.101925

[B7] Budzys A. Kurasova O. Medvedev V. (2025). Integrating deep learning and data fusion for advanced keystroke dynamics authentication. Comput. Stand. Interf. 92:103931. doi: 10.1016/j.csi.2024.103931

[B8] Chang H. C. Li J. Stamp M. (2022). “Machine learning-based analysis of free-text keystroke dynamics,” in Artificial Intelligence for Cybersecurity, eds. M. Stamp, C. A. Visaggio, F. Mercaldo, and F. Di Troia (Advances in Information Security), 331–356. doi: 10.1007/978-3-030-97087-1_14

[B9] Chen T. Guestrin C. (2016). “XGBoost: a scalable tree boosting system,” in Proceedings of the ACM SIGKDD international conference on knowledge discovery and data mining, 785–794. doi: 10.1145/2939672.2939785

[B10] Choi M. Lee S. Jo M. Shin J. S. (2021). Keystroke dynamics-based authentication using unique keypad. Sensors 21:2242. doi: 10.3390/s21062242 33806907 PMC8004962

[B11] Costa J. C. Roxo T. Proença H. Inácio P. R. M. (2024). How deep learning sees the world: a survey on adversarial attacks and defenses. IEEE Access 12, 61113–61136. doi: 10.1109/ACCESS.2024.3395118

[B12] Crawford H. (2010). “Keystroke dynamics: characteristics and opportunities,” in Proceedings of the eighth international conference on privacy, security and trust, Ottawa, Canada, 205–212. doi: 10.1109/PST.2010.5593258

[B13] Darabseh A. Pal D. (2020). “Performance analysis of keystroke dynamics using classification algorithms,” in Proceedings of the third international conference on information and computer technologies, San Jose, CA, USA, 124–130. doi: 10.1109/ICICT50521.2020.00027

[B14] Dias T. Vitorino J. Maia E. Sousa O. Praça I. (2023). KeyRecs: a keystroke dynamics and typing pattern recognition dataset. Data Brief 50:109509. doi: 10.1016/j.dib.2023.109509 37663780 PMC10474054

[B15] Giot R. El-Abed M. Rosenberger C. (2009). “GREYC keystroke: a benchmark for keystroke dynamics biometric systems,” in Proceedings of the IEEE international conference on biometrics: theory, applications and systems (BTAS), Washington, DC, USA, 1–6. doi: 10.1109/BTAS.2009.5339051

[B16] Grunova D. Tsimperidis I. (2023). Finding the age and education level of Bulgarian-speaking internet users using keystroke dynamics. Eng—Adv. Eng. 4, 2711–2721. doi: 10.3390/eng4040154

[B17] Gumelar A. B. (2019). “An anatomy of machine learning data visualization,” in Proceedings of the international seminar on application for technology of information and communication, 1–6. doi: 10.1109/ISEMANTIC.2019.8884340

[B18] IBM (2024). Security analysis of cryptographic algorithms. Available online at: https://www.ibm.com/reports/data-breach (Accessed August 25, 2024).

[B19] Jawed H. Ziad Z. Khan M. M. Asrar M. (2018). Anomaly detection through keystroke and tap dynamics implemented via machine learning algorithms. Turkish J. Electr. Eng. Comput. Sci. 26, 1698–1709. doi: 10.3906/elk-1711-410

[B20] Kaur D. Carvalho G. H. Dividino R. Woungang I. Anpalagan A. (2026). Novel explainable CNN-LightGBM model for smartphone continuous authentication. IEEE Access 14, 45826–45843. doi: 10.1109/ACCESS.2026.3672372

[B21] Killourhy K. S. Maxion R. A. (2009). “Comparing anomaly-detection algorithms for keystroke dynamics,” in Proceedings of the IEEE/IFIP international conference on dependable systems and networks, Lisbon, Portugal, 125–134. doi: 10.1109/DSN.2009.5270346

[B22] Kim D. I. Lee S. Shin J. S. (2020). A new feature scoring method in keystroke dynamics-based user authentications. IEEE Access 8, 27901–27914. doi: 10.1109/ACCESS.2020.2968918

[B23] Kim J. Kang P. (2020). Freely typed keystroke dynamics-based user authentication for mobile devices based on heterogeneous features. Pattern Recognit. 108:107556. doi: 10.1016/j.patcog.2020.107556

[B24] Kulkarni V. Y. Sinha P. K. (2012). “Pruning of random forest classifiers: a survey and future directions,” in Proceedings of the international conference on data science and engineering, 64–68. doi: 10.1109/ICDSE.2012.6282329

[B25] Loy C. C. Lai W. K. Tse C. K. (2007). Keystroke patterns classification from free text input and application to novel user classification problems. Pattern Recognit. 40, 3396–3406. Available online at: https://personal.ie.cuhk.edu.hk/~ccloy/downloads_keystroke100.html

[B26] Maalej A. Kallel I. S1nchez M. J. (2020). Emosurv: a typing biometric (keystroke dynamics) dataset with emotion labels created using computer keyboards. IEEE Dataport. doi: 10.21227/eae6-pk42

[B27] Maharana K. Mondal S. Nemade B. (2022). A review: Data pre-processing and data augmentation techniques. Global Trans. Proc. 3, 91–99. doi: 10.1016/j.gltp.2022.04.020

[B28] Martins A. Dias T. Dias A. Vitorino J. Maia E. Praça I. (2025). Keystroke dynamics for intelligent biometric authentication with machine learning. Disc. Appl. Sci. 7:992. doi: 10.1007/s42452-025-07449-5

[B29] Medvedev V. Budzys A. Kurasova O. (2025). A decision-making framework for user authentication using keystroke dynamics. Comput. Secur. 155:104494. doi: 10.1016/j.cose.2025.104494

[B30] Mhenni A. Cherrier E. Rosenberger C. Amara N. E. B. (2019). Double serial adaptation mechanism for keystroke dynamics authentication based on a single password. Comput. Secur. 83, 151–166. doi: 10.1016/j.cose.2019.02.002

[B31] Mienye I. D. Sun Y. (2022). A survey of ensemble learning: concepts, algorithms, applications, and prospects. IEEE Access 10, 99129–99149. doi: 10.1109/ACCESS.2022.3207287

[B32] Miller C. Portlock T. Nyaga D. M. O'Sullivan J. M. (2024). A review of model evaluation metrics for machine learning in genetics and genomics. Front. Bioinform. 4:1457619. doi: 10.3389/fbinf.2024.1457619 39318760 PMC11420621

[B33] Mokoena T. Sabatta D. (2020). “User classification by keystroke dynamics using text retrieval methods,” in Proceedings of the international SAUPEC/RobMech/PRASA conference, Cape Town, South Africa, 1–6. doi: 10.1109/SAUPEC/RobMech/PRASA48453.2020.9040956

[B34] Monaco J. V. Bakelman N. Cha S. H. Tappert C. C. (2013). “Recent advances in the development of a long-text-input keystroke biometric authentication system for arbitrary text input,” in Proceedings of the IEEE international conference on technologies for homeland security (HST), Waltham, MA, USA, 312–317. doi: 10.1109/EISIC.2013.16

[B35] Muliono Y. Ham H. Darmawan D. (2018). Keystroke dynamic classification using machine learning for password authorization. Proc. Comput. Sci. 135, 564–569. doi: 10.1016/j.procs.2018.08.209

[B36] Patil R. A. Renke A. L. (2016). Keystroke dynamics for user authentication and identification by using typing rhythm. Int. J. Comput. Applic. 144, 27–33. doi: 10.5120/ijca2016910432

[B37] Piugie Y. B. W. Di Manno J. Rosenberger C. Charrier C. (2022). “Keystroke dynamics based user authentication using deep learning neural networks,” in Proceedings of the international conference on cyberworlds, Kanazawa, Japan, 220–227. doi: 10.1109/CW55638.2022.00052

[B38] Porwik P. Doroz R. Wesolowski T. E. (2020). Dynamic keystroke pattern analysis and classifiers with competence for user recognition. Appl. Soft Comput. 99:106902. doi: 10.1016/j.asoc.2020.106902

[B39] Quimatio B. M. A. Njike O. F. Y. Nkenlifack M. (2022). “User authentication through keystroke dynamics based on ensemble learning approach,” in Proceedings of the colloque Africain sur la recherche en informatique et en mathématiques appliquées (CARI).

[B40] Radosevich D. Borowczak M. (2026). A statistical approach for keystroke continuous authentication subversion. IEEE Access 14, 20694–20704. doi: 10.1109/ACCESS.2026.3662283

[B41] Rashedi K. A. Ismail M. T. Wadi S. A. Serroukh A. Alshammari T. S. Jaber J. J. (2024). Multi-layer perceptron-based classification with application to outlier detection in Saudi Arabia stock returns. J. Risk Financ. Manag. 17:69. doi: 10.3390/jrfm17020069

[B42] Rawat T. Khemchandani V. (2017). Feature engineering tools and techniques for better classification performance. Int. J. Innov. Eng. Technol. 8, 169–179. doi: 10.21172/ijiet.82.024

[B43] Risto H. N. Graven O. H. (2024). Fixed-text keystroke dynamics authentication data set—collection and analysis. Ann. Telecommun. 79, 731–743. doi: 10.1007/s12243-024-01039-z

[B44] Sahu C. Banavar M. Schuckers S. (2020). “A novel distance-based algorithm for multi-user classification in keystroke dynamics,” in Proceedings of the asilomar conference on signals, systems and computers, 63–67. doi: 10.1109/IEEECONF51394.2020.9443407

[B45] Salem A. Sharieh A. Sleit A. Jabri R. (2019). Enhanced authentication system performance based on keystroke dynamics using classification algorithms. KSII Trans. Internet Inf. Syst. 13:4076. doi: 10.3837/tiis.2019.08.014

[B46] Shadman R. Wahab A. A. Manno M. Lukaszewski M. Hou D. Hussain F. (2025). Keystroke dynamics: concepts, techniques, and applications. ACM Comput. Surv. 57, 1–35. doi: 10.1145/3733103

[B47] Shenoy K. (2024). Keystroke dynamics challenge 1. Available online at: https://www.kaggle.com/competitions/keystroke-dynamics-challenge-1/data (Accessed December, 2024).

[B48] Sitová Z. Sedĕnka J. Yang Q. Peng G. Zhou G. Gasti P. . (2016). HMOG: new behavioral biometric features for continuous authentication of smartphone users. IEEE Trans. Inf. Forens. Secur. 11, 877–892. doi: 10.1109/TIFS.2015.2506542

[B49] Sun Y. Ceker H. Upadhyaya S. (2016). “Shared keystroke dataset for continuous authentication,” in Proceedings of the IEEE international workshop on information forensics and security (WIFS), Abu Dhabi, United Arab Emirates, 1–6. doi: 10.1109/WIFS.2016.7823894

[B50] Tsai C.-J. Shih K.-J. (2019). Mining a new biometrics to improve the accuracy of keystroke dynamics-based authentication system on free-text. Appl. Soft Comput. 80, 125–137. doi: 10.1016/j.asoc.2019.03.033

[B51] Tsimperidis I. Arampatzis A. Karakos A. (2018). Keystroke dynamics features for gender recognition. Dig. Investig. 24, 4–10. doi: 10.1016/j.diin.2018.01.018

[B52] Tsimperidis I. Asvesta O. D. Vrochidou E. Papakostas G. A. (2024). IKDD: a keystroke dynamics dataset for user classification. Information 15:511. doi: 10.3390/info15090511

[B53] Tsimperidis I. Peikos G. Arampatzis A. (2021a). “Classifying users through keystroke dynamics,” in Studies in classification, data analysis, and knowledge organization, 311–319. doi: 10.1007/978-3-030-60104-1_34

[B54] Tsimperidis I. Yucel C. Katos V. (2021b). Age and gender as cyber attribution features in keystroke dynamic-based user classification processes. Electronics 10:835. doi: 10.3390/electronics10070835

[B55] Turner C. Anthony A. J. Aksu M. Langdond H. (2011). The wavelet and Fourier transforms in feature extraction for text-dependent, filterbank-based speaker recognition. Procedia Comput. Sci. 6, 124–129. doi: 10.1016/j.procs.2011.08.024

[B56] Verdonck T. Baesens B. Óskarsdóttir M. Vanden Broucke S. (2021). Special issue on feature engineering editorial. Mach. Learn. 113, 3917–3928. doi: 10.1007/s10994-021-06042-2

[B57] Wang X. Shi Y. Zheng K. Zhang Y. Hong W. Cao S. (2022). User authentication method based on keystroke dynamics and mouse dynamics with scene-irrelevant features in hybrid scenes. Sensors 22:6627. doi: 10.3390/s22176627 36081085 PMC9460698

[B58] Wong T.-T. (2015). Performance evaluation of classification algorithms by k-fold and leave-one-out cross validation. Pattern Recognit. 48, 2839–2846. doi: 10.1016/j.patcog.2015.03.009

[B59] Wu T. Zheng K. Wu C. Wang X. Xu G. (2019). “User identification by keystroke dynamics based on feature correlation analysis and feature optimization,” in Proceedings of the IEEE international conference on computer and communications, Chengdu, China, 40–46. doi: 10.1109/ICCC47050.2019.9064347

[B60] Yang Y. Guo B. Liang Y. Zhao K. Yu Z. (2024). Cross-device free-text keystroke dynamics authentication using federated learning. Personal Ubiquit. Comput. 28, 491–505. doi: 10.1007/s00779-024-01832-6

[B61] Zhong Y. Deng Y. Jain A. K. (2012). “Keystroke dynamics for user authentication,” in Proceedings of the IEEE computer society conference on computer vision and pattern recognition workshops. doi: 10.1109/CVPRW.2012.6239225

